# The Presidency, and Place of Meeting of the American Medical Association

**Published:** 1857-04

**Authors:** 


### *The Presidency, and place of meeting of the American Medical Association.*

No member of the profession who has any regard for the honor and interest of medical science in this country, can regard with any other than feelings of the loftiest pride, the achievements and position of our National Medical Organization. It has been in existence but a few years, not having yet attained its majority, and yet so much has already been accomplished by it, that we have just reason to be proud of our Association. We are aware that there are defects in its organization, but it does not follow that they may not, or that they will not be removed by time.

We are also aware that some members of the profession expect *too much* from the Association—that they look for its positive action in the correction of abuses which it can only reach by a sort of moral influence. The Association is not a law making power for the profession at large. It has a code of ethics for the government of *its members,* and all the power it has, is, to cut them off from membership for non-conformity to its code. This it has done, and this we trust it will always do, whenever occasion requires.

Among the more prominent defects in the machinery of the Association hitherto, is the law ordained by custom, that the President of the Association should be chosen from among the profession of the city in which its meetings is held. This policy may be said to have been inaugurated at the second annual meeting of the Association held in Boston, in 1849. Dr. Jonathan Knight, of New Haven, was President of the preliminary Convention held in New York, in 1846, and in the Convention at which the Association was formed, held in Philadelphia in 1847, Dr. Nathaniel, was chosen its first President. Although Dr. Chapman was a resident of that city, we doubt whether any one will question the propriety of this choice, as he unquestionably stood at the head of the profession in the country. At the first annual meeting held in Baltimore, the President chosen—Dr. A. H. Stevens—was resident of New York. So that, in reality, the policy of choosing the President from among the profession of the city where the Association meets, was inaugurated in Boston, when Dr. John C. Warren was chosen. We must not be understood to complain of the propriety of the choice in any of these cases, for all the distinguished men who have occupied the post in question, were eminently worthy of the honor, under the rule by which they were elected. But with Dr. Hunt, of the *Buffalo Journal,* we think the time has come when the Presidency of the American Medical Association should not be measured by any local considerations, or bound by precedent, but should be an honor conferred by the profession of a nation, on its most distinguished member, whether he be a resident of the city or State where the meeting is held or not. We are glad to see that the medical press, and the profession generally, are ripe for a return to the original order of things, and it is especially honorable to the profession of Tennessee, the State in which the next meeting is to be held—for we have no doubt but the *Nashville Journal* represents the sentiments of the profession of that State—that they have come forward with a commendable eagerness, to have the proposed change made at the ensuing meeting at Nashville. The Presidency of our National Medical Association should be regarded as a mark of distinction conferred by the profession of a nation on one of its members, in gratitude for actual service rendered to the cause of medical science, and to the interests of the Association, whether he be a resident of a large city, or of some country hamlet.

With regard to the place of meeting of the Association, we think that the time approaches when the migratory character of the meetings should be in part, at least, done away with. We think that, so far, the plan has been a wise one, and when the Association shall have had one session in the southwestern section of the country, when the profession of all sections of the Union, except the extreme West, will have had an opportunity of forming its acquaintance, as it were, some proper place should be chosen, where most of its sessions should be held.

Washington has been proposed as the most proper place, and it strikes us as eminently so, particularly if, as we think is possible, some arrangement can be made by which apartments in the Smithsonian Institution could be devoted to its uses. We would not have every session of the Association held at one point, but would have at least one out of every three held in other parts of the country. A writer in the *Peninsular Journal of Medicine* proposes that every third meeting should be held in Philadelphia, but we should prefer that they be held at the seat of the national government. We hope this question will come up for discussion at Nashville.—*Medical & Surgical Reporter.*

